# Splenocytes derived from young WT mice prevent AD progression in APPswe/PSENldE9 transgenic mice

**DOI:** 10.18632/oncotarget.4930

**Published:** 2015-08-06

**Authors:** Fei Wang, Xueyan Shen, Shuping Li, Long Chen, Yanru Wang, Jie Qin, Guomin Zhou, Yuwen Peng, Xiaoyuan Feng, Ruixi Li, Chunmin Liang

**Affiliations:** ^1^ Department of Anatomy and Histology & Embryology, Shanghai Medical College of Fudan University, Shanghai, P. R. China; ^2^ Department of Radiology, Huashan Hospital, Fudan University, Shanghai, P. R. China; ^3^ Department of Radiology, PLA No. 455 Hospital, Shanghai, P. R. China

**Keywords:** Gerotarget, Alzheimer's disease, splenocytes, immunosenescence, GDF11, Treg

## Abstract

Immunosenescence contributes to pathogenesis of Alzheimer's disease (AD) in the elderly. In this study, we explored the effects of young wild type (WT) splenocytes (ySCs) on Alzheimer's disease by transplanting ySCs into APPswe/PSENldE9 transgenic mice. Young WT splenocytes not only prevented AD, but also improved the spatial learning and memory of APPswe/PSENldE9 transgenic mice. Young WT splenocytes enhanced Aβ clearance, decreased astrogliosis and increased systemic growth differentiation factor 11 (GDF11) levels. Splenocytes derived from old AD mouse promoted AD. There was an increased number of regulatory T cells (Tregs) among old AD splenocytes. We suggest that alterations of GDF11 and Tregs are involved in AD progression and that rejuvenation of the immune system is a potential therapeutic strategy in AD.

## INTRODUCTION

Alzheimer's disease (AD) is an age-related neurodegenerative disorder characterized by progressive memory loss and cognitive decline. Immune dysfunction is involved in the pathogenesis of AD [1–5], therefore understanding and correcting this dysfunction should help in the prevention of this disease.

Immune cells such as microglia and T cells, as well as various cytokines, and other immune factors, play an important role in maintaining the normal microenvironment in the Central Nervous System (CNS) and contribute to brain health and function. These cells may prevent AD and other neurodegenerative diseases [6–10]. Recent studies place immunosenescence with ageing at the center of AD progression [11–15]. Aging related microglia dysfunction and over-inflammation are involved in AD pathogenesis and affect AD therapy [1, 4, 15, 16]. Therefore, reversing immunosenescence to restore immune protection in the CNS is a promising strategy for AD therapy. Some studies have reported that transplantation of bone marrow derived- microglia or Aβ specific T cells could effectively prevent the pathological progression and cognitive decline of AD [3, 17, 18]. Inhibition of mTOR, an anti-aging strategy, ameliorates immunosenescence in old humans, and could also suppress brain aging to prevent neurodegeneration [19–23].

Recent research has shown that young blood can reverse cognitive impairments and improve cognitive function by regulating related cytokines, such as Growth Differentiation Factor 11 (GDF11) and C-C motif Chemokine 11 (CCL11) [16, 24, 25]. Moreover, transplanting bone marrow derived- microglia, or Aβ specific T cells, can ameliorate cerebral microglial function and effectively prevent the pathological progression and cognitive decline of AD [3, 17, 18]. We found that a combined treatment of Aβ_1–42_-BMDCs (bone marrow derived dendritic cell) plus splenocytes from young mice could prevent AD progression by restoring the function of senescent microglia [26]. It appears that immune cells from young healthy bodies are able to restore immune protection in the CNS of AD patients and prevent AD progression [24, 27], therefore we propose that rejuvenation factors, such as GDF11, may be directly involved in restoring immune protection.

We investigated the effects of transplanting splenocytes, derived from young WT mice, into a transgenic mouse model of Alzheimer's disease, APPswe/PSENldE9. We also transplanted splenocytes, derived from AD mice, to investigate the effects on AD pathogenesis.

## RESULTS

### Splenocytes from young WT mice reduced cerebral Aβ burden in AD mice

**Figure 1:**
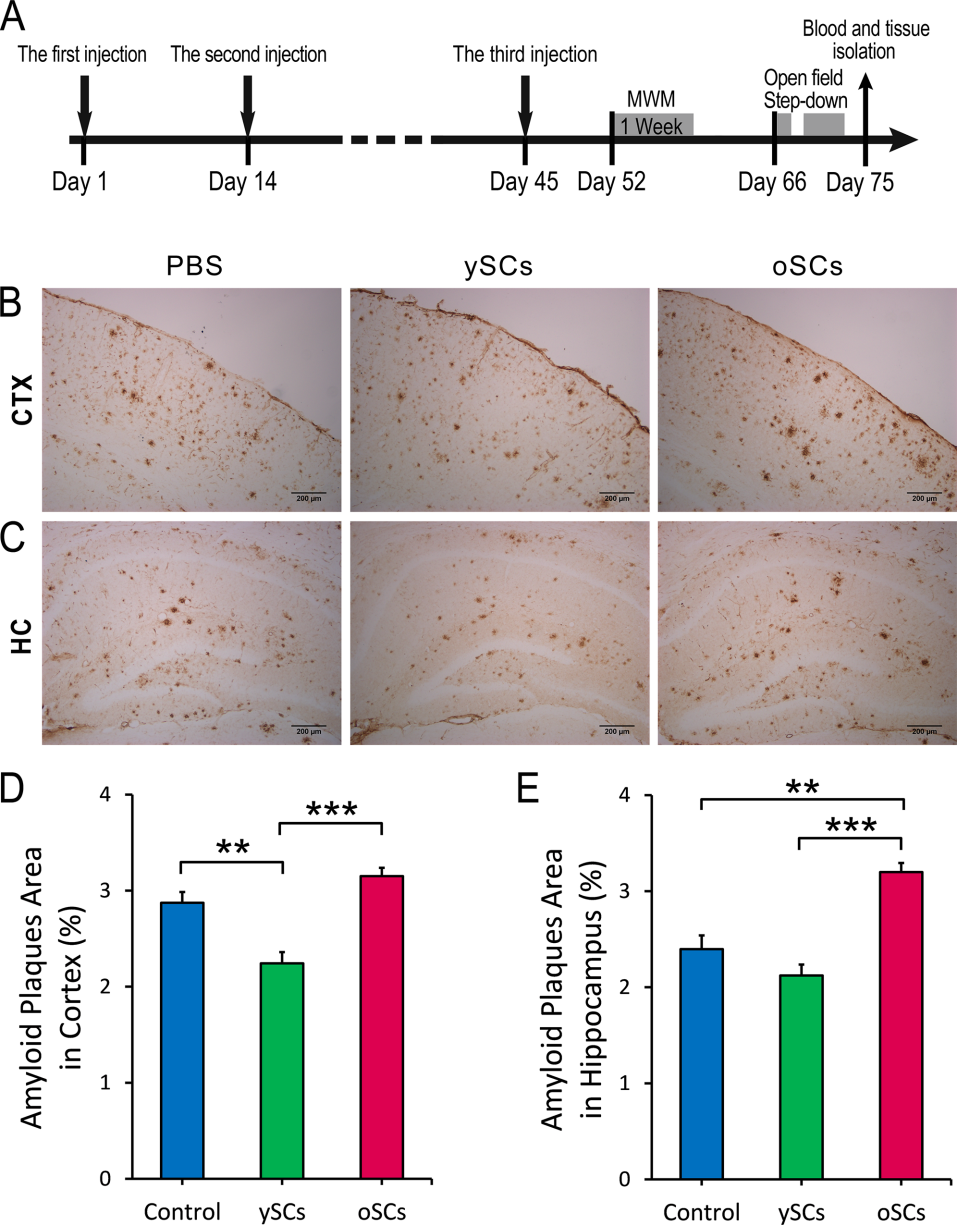
The effect of SCs treatments on cerebral Aβ clearance **A.** Experimental scheme. Aged APPswe/PSENldE9 transgenic mice (14–15 months) received 3 times intraperitoneal injections of splenocytes (young WT mice or old APPswe/PSENldE9 transgenic mice) or 0.01 M PBS on Day 1, Day 14, and Day 45. After administration, mice were performed Morris water maze (MWM) (from Day 52 to Day 58), Open field (on Day 66) and Step down (from Day 69 to Day 71), and sacrificed on Day 75. **B.** and **C.** The Aβ burden was measured by IHC of serials brain section in cortex (CTX) and hippocampus (HC). **D.** and **E.** The percentage of Aβ plaques area of brain section (8–10 sections per mouse) in cortex and hippocampus. Data are presented as mean ± SEM, *n* = 6. **p < 0.01, ***p < 0.001), One-Way ANOVA with LSD post-hoc test. Scale bar, 200 μm.

Changes in Aβ burden in cerebral amyloid plaques were detected by immunochemistry in old APPswe/PSENldE9 transgenic mice treated with splenocytes derived from young WT mice or old transgenic mice. Figure 1B and 1D show that treatment with ySCs (splenocytes derived from young WT mice) reduced Aβ plaques in the cortex compared with a PBS control (One-Way ANOVA with LSD post-hoc test, *F*_(2, 36)_ = 25.620, *p* = 0.002). Moreover, treatment with ySCs decreased Aβ plaques in both cortex and hippocampus in contrast to treatment with oSCs (splenocytes derived from old transgenic mice) (One-Way ANOVA with LSD post-hoc test, *F*_(2, 13)_ = 19.486; *p* < 0.001; Figure 1B–1E). Treatment with oSCs only increased Aβ plaques in the hippocampus compared with control (One-Way ANOVA with LSD post-hoc test, *F*_(2, 13)_ = 19.486; *p* = 0.001; Figure 1C and 1E).

### Splenocytes from young WT mice modulated astrocytic response in AD mice

**Figure 2:**
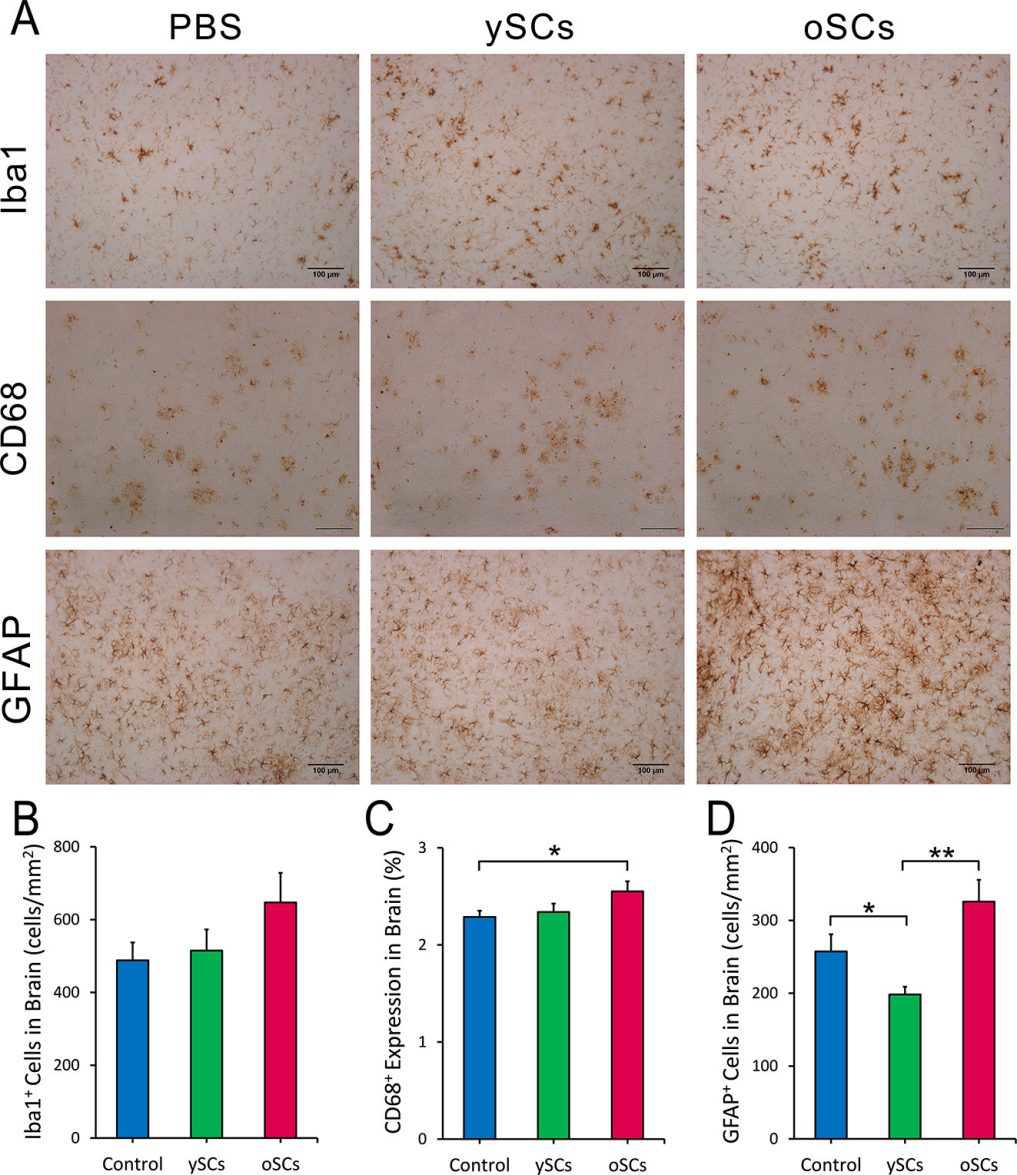
Microglia and astrocyte response after SCs treatments **A.** Representative images of microglia (Iba1), CD68 positive microglia and astrocytes (GFAP) in the brain of cortex stained by IHC after administration. **B.** The number of Iba1 positive microglia per mm^2^ on the brain sections was quantified and analyzed in APPswe/PSENldE9 transgenic mice with different treatments. **C.** The percentage of CD68 covered area on brain sections were used for analysis. **D.** The number of GFAP positive astrocytes per mm^2^ on the brain sections was analyzed. For all the statistical analysis, the regions of cortex and hippocampus were selected by NIH image J (B–D). Date are presented as mean ± SEM, *n* = 6. *p < 0.05, **p < 0.01, one way ANOVA with LSD post-hoc test. Scale bar, 100 μm.

The response of cerebral microglia and astrocytes is related to the accumulation of Aβ and AD pathogenesis. Therefore, we used immunohistochemistry to analyze Iba1^+^ microglia and GFAP^+^ astrocytes after splenocyte administration. Figure 2 shows that both ySCs and oSCs treatment did not affect the number of Iba1^+^ microglia in the brain of old APPswe/PSENldE9 mice, whereas oSCs treatment increased cerebral CD68 expression in the AD mice compared with PBS control (One-Way ANOVA with LSD post-hoc test, *F*_(2, 67)_ = 2.950; *p* = 0.020). Concurrently, we found that the ySCs treatment decreased the number of GFAP^+^ astrocytes in comparison with PBS control (One-Way ANOVA with LSD post-hoc test, *F*_(2, 51)_ = 7.325; *p* = 0.046), whereas the oSCs treatment increased the number of GFAP^+^ astrocytes in comparison with ySCs treatment (One-Way ANOVA with LSD post-hoc test, *F*_(2, 51)_ = 7.325; *p* = 0.046; Figure 2A, 2D).

### Splenocytes from young WT mice slowed learning and memory deficits in AD mice

**Figure 3:**
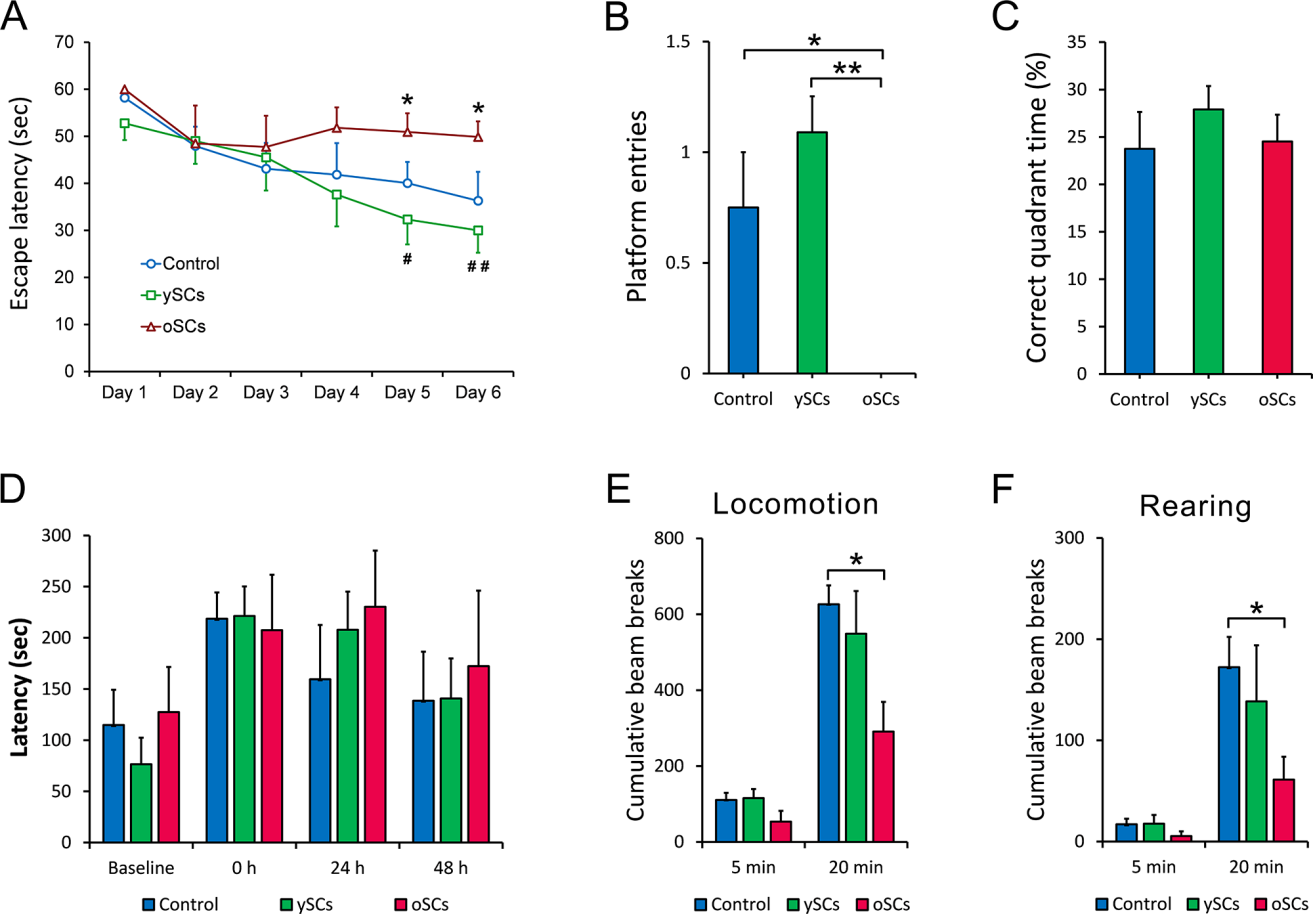
Behavioral test One week after the last injection, all mice were performed Morris Water Maze test, then Open filed test and Step-down test two week later. **A.** The escape latency in 6 days was measured and analyzed. The oSCs treatment (*n* = 6) increased the escape latency of APPswe/PSENldE9 transgenic mice at day 5 and day 6 compared with PBS Control (*n* = 6) (*p < 0.05). The ySCs treatment (*n* = 6) showed a decreased escape latency at day 5 (^#^*p* < 0.05) and day 6 (^##^*p* < 0.01), versus oSCs treatment (Repeated-measures analysis of variance (ANOVA), followed by Fisher's least significant difference post-hoc test). **B.** and **C.** The number of platform entries and the percentage of time of correct quadrant (platform located) were measured in the test at day 7. One way ANOVA with LSD post-hoc test was used for statistical analysis, *p < 0.05, **p < 0.01. **D.** The latency to step-down was assessed in step-down test. There was no difference in the training (0 hours) and 24, 48 hours after training in all groups (Kruskal-Wallis non-parametric ANOVA). **E.** and **F.** Locomotion and rearing in 5 and 20 minutes were measured for statistical analysis in open field test (One way ANOVA with LSD post-hoc test, *p < 0.05). The number of beam break was used to quantify the locomotion and rearing. Data are presented as mean ± SEM, *n* = 6.

We used the Morris Water Maze (MWM) to measure the effects of different treatments on spatial learning and memory. Shown in the training and spatial probe test (Figure 3A, 3B), ySCs treatment on old AD mice improved cognitive impairments compared with the oSCs treatment (in the escape latency: Repeated-measures analysis of variance (ANOVA), followed by Fisher's least significant difference post-hoc test, *F*_(2, 20)_ = 2.798, *p* = 0.073; platform entries: One way ANOVA with LSD post-hoc test, *F*_(2, 20)_ = 2.696, *p* = 0.145; Kruskal-Wallis nonparametric ANOVA, *p* = 0.811), though it could not improve spatial learning and memory compared with PBS control (Repeated-measures analysis of variance (ANOVA), followed by Fisher's least significant difference post-hoc test, *F*_(2, 20)_ = 2.696, *p* = 0.039; platform entries: One way ANOVA with LSD post-hoc test, *F*_(2, 20)_ = 2.798, *p* = 0.004, Kruskal-Wallis nonparametric ANOVA, *p* = 0.014). However, the oSCs treatment accelerated cognitive impairment on spatial learning and memory, as shown by an increased escape latency response (Repeated-measures analysis of variance (ANOVA), followed by Fisher's least significant difference post-hoc test, *F*_(2, 20)_ = 2.696, *p* = 0.008) and decreased platform entries in the spatial probe test compared with PBS control (One-Way ANOVA with LSD post-hoc test, *F*_(2, 20)_ = 2.798; *p* = 0.043; Kruskal-Wallis nonparametric ANOVA, *p* = 0.099). There was no significant difference in time spent within the correct quadrant among different groups (Figure 3C).

We also used step-down and open field tasks to assess behavioral deficits. There was no difference in the step-down test among different treatment groups (Figure 3D). Treatment with oSCs decreased time in the 20-minute locomotion, open-field task (One-Way ANOVA with LSD post-hoc test, *F*_(2, 20)_ = 1.914, *p* = 0.034; Kruskal-Wallis nonparametric ANOVA, *p* = 0.010) and rearing (One way ANOVA with LSD post-hoc test, *F*_(2, 20)_ = 0.837, *p* = 0.041; Kruskal-Wallis nonparametric ANOVA, *p* = 0.021) of old AD mice in open field compared with control. Treatment with ySCs did not affect the locomotion and rearing of old AD mice (Figure 3E, 3F).

**Figure 4:**
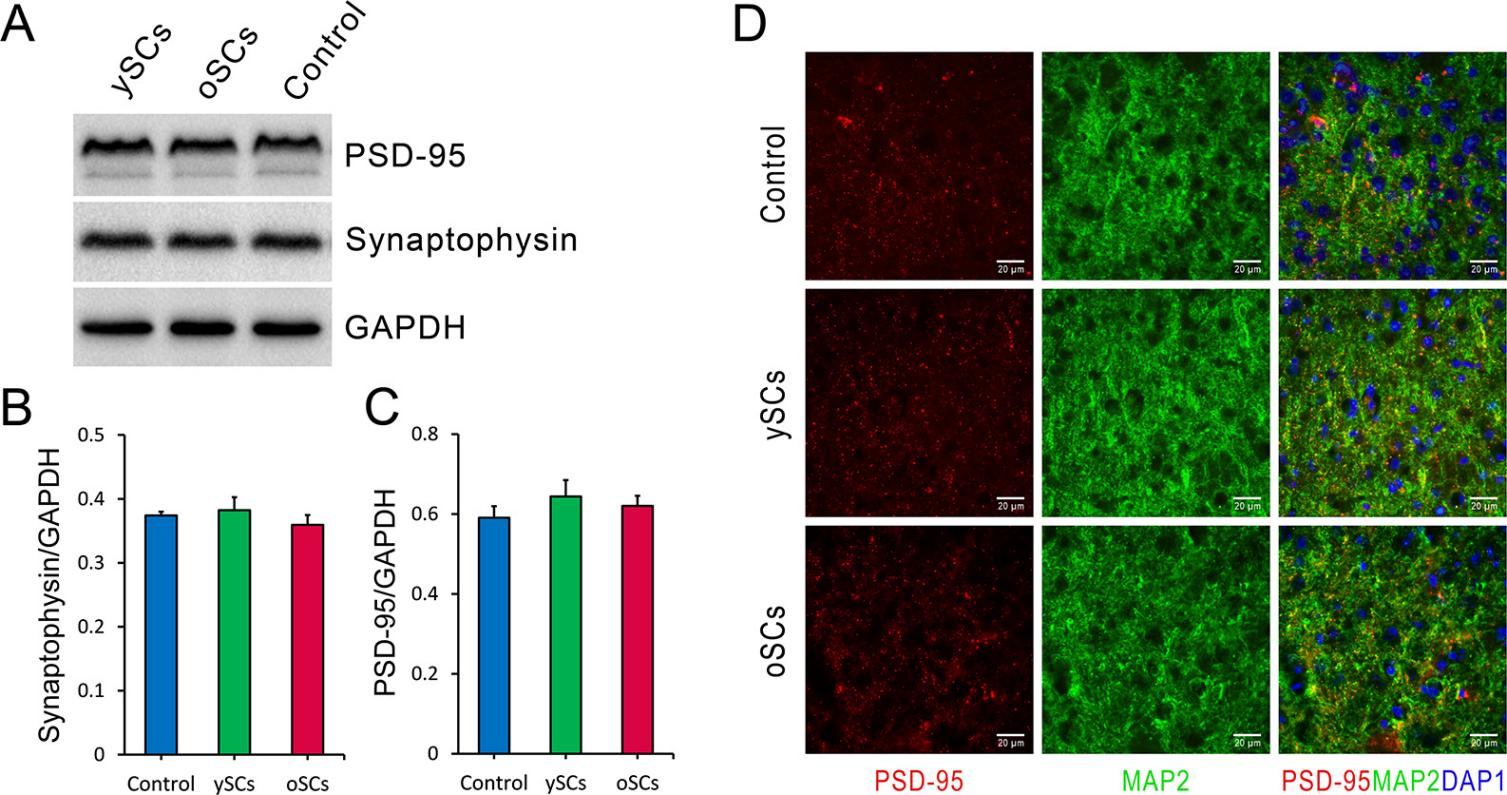
The effect of SCs treatments on cerebral synaptic plasticity of old APPswe/PSENldE9 transgenic mice **A.** After administration, the levels of postsynaptic density protein 95 (PSD-95) and synaptophysin, synaptic markers, of brain homogenization were detected by Western Blot after SCs treatments. GAPDH was used as loading control. **B.** and **C.** The levels of PSD-95 and synaptophysin normalized to GAPDH were used for statistical analysis. There was no difference of PSD-95 and synaptophysin expression of brain in the three groups with various treatments. **D.** Cerebral synapses were labeled by staining postsynaptic density protein 95 (PSD-95, red) and microtubule associated protein 2 (MAP2, green) after SCs treatments. Data are presented as mean ± SEM, *n* = 6. One way ANOVA with LSD post-hoc test was used for statistical analysis. Scale bar, 20 μm.

Synapse loss correlates to cognitive deterioration in AD [28], therefore we used Western blots to measure levels of PSD-95 (postsynaptic density protein 95) and synaptophysin in brain tissues after splenocyte administration. We did not find any difference in PSD-95 (One-Way ANOVA with LSD post-hoc test, *F*_(5, 42)_ = 0.337, *p* = 0.888) or synaptophysin (One-Way ANOVA with LSD post-hoc test, *F*_(5, 54)_ = 1.813, *p* = 0.126) between treatment groups (Figure 4A–4C). We also used immunofluorescence to examine neurons and synapses in both the cortex and hippocampus (Figure 4D). We did not notice any obvious changes in synapse morphology or density in both splenocyte treatment groups compared to control.

### Increased frequency of Treg cells in old mice derived splenocytes

**Figure 5:**
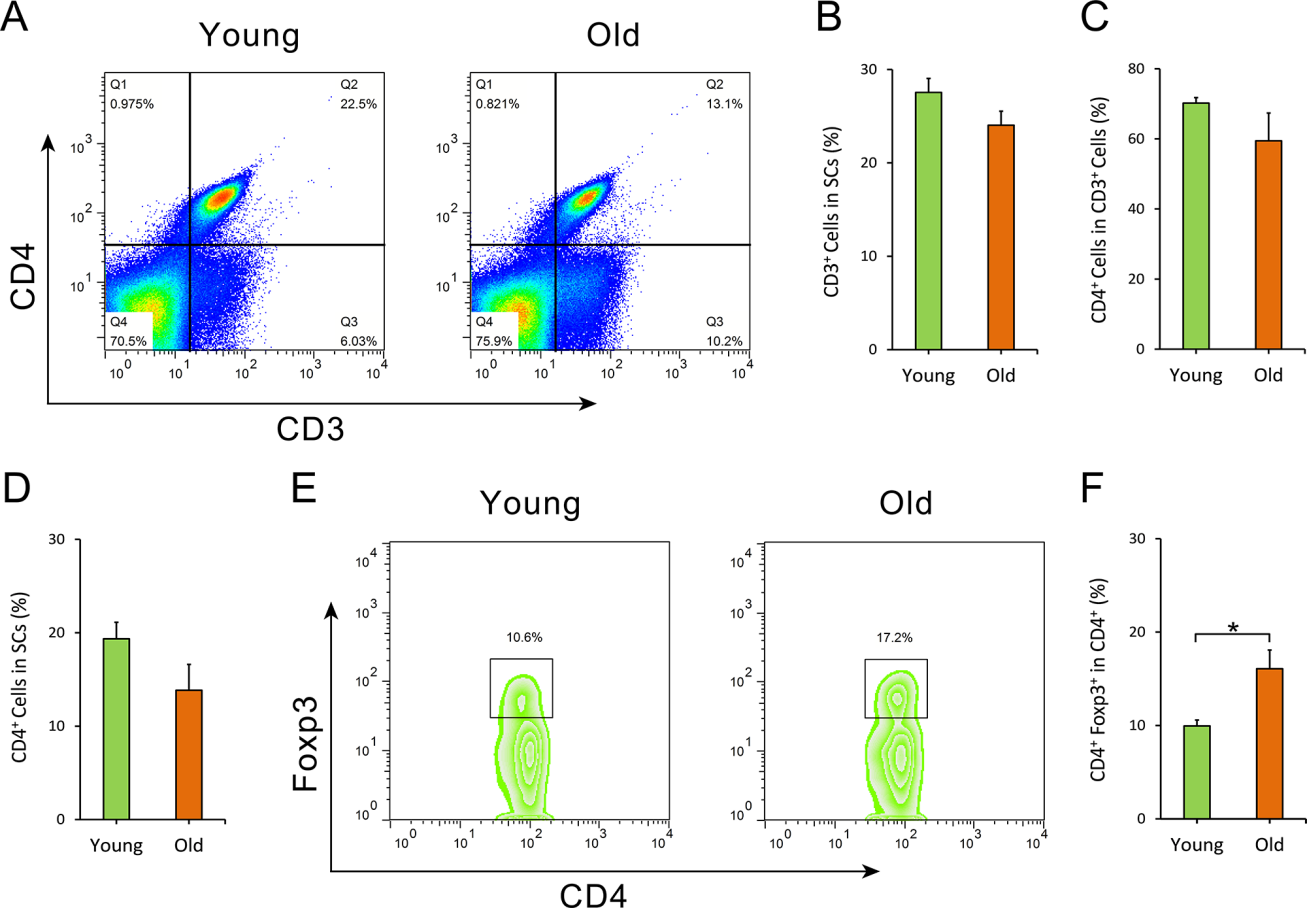
Flow cytometry analysis of splenocytes The frequency of CD3^+^ T cells, CD4^+^ T cells and CD4^+^ Foxp3^+^ Treg cells of splenocytes from both old APPswe/PSENldE9 transgenic mice (old, 14–15 month-old) and young mice (young, 6–8 week-old) were analyzed by flow cytometry. **A.** Dot plots of CD3^+^ T cells, CD4^+^ T cells in splenocytes. **B.** Statistical analysis for the percentage of CD3^+^ T cells in splenocytes. **C.** The percentage of CD4^+^ T cells in CD3^+^ T cells of SCs isolated from old AD mice and young WT mice. **D.** The percentage of CD3^+^ CD4^+^ T cells in splenocytes. **E.** Representative flow cytometry of CD4^+^ Foxp3^+^ Treg cells within CD4^+^ T lymphocytes. **F.** Statistical analysis for the frequency of CD4^+^ Foxp3^+^ Treg cells in CD4^+^ T cells. Data are presented as mean ± SEM, *n* = 6. *p < 0.05, Student's *t*-test.

It has been reported that T cells, including CD4^+^ T cells and Treg cells, prevent AD pathogenesis and imorove cognitive function in AD mice. We analyzed the subsets of these splenocytes with flow cytometry. The results showed no difference in frequency of CD3^+^ T cells (Student's *t*-test, *t* = 1.718, df = 5, *p* = 0.146) and CD4^+^ T cells (Student's *t*-test, *t* = 1.764, df = 5, *p* = 0.138) in the total splenocytes derived from young WT mice and old AD mice (Figure 5A–5D), however, it was noted that there was a higher frequency of Tregs in oSCs (Student's *t*-test, *t* = 2.272, df = 5, *p* = 0.028) (Figure 5E, 5F).

### Splenocytes from young WT mice increased systemic GDF11 in AD mice

**Figure 6:**
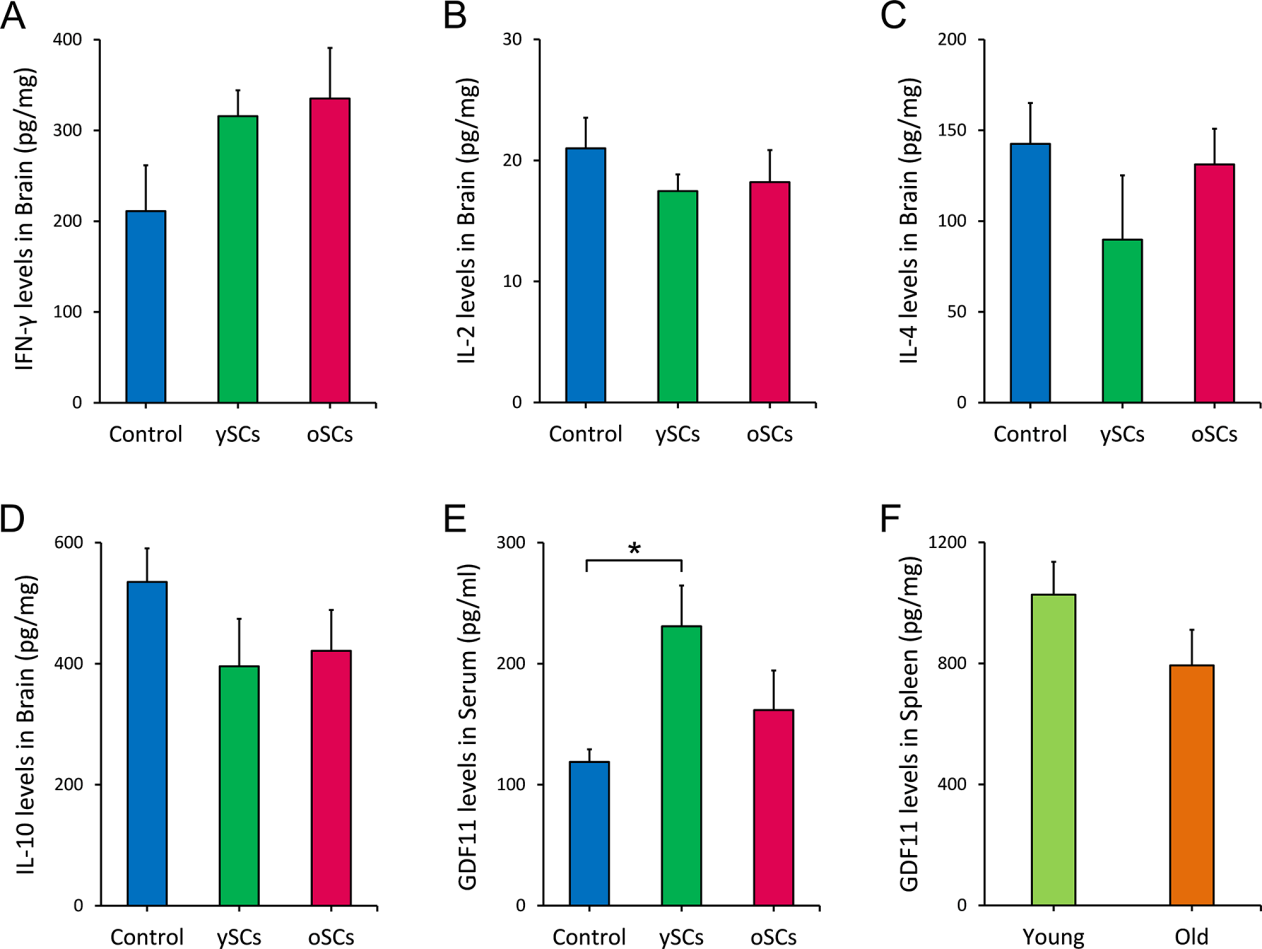
The ySCs treatment increased GDF11 levels in blood of APPswe/PSENldE9 transgenic mice **A-D.** After SCs treatment, the levels of IFN-γ, IL-2, IL-4, IL-10 in brain of APPswe/PSENldE9 transgenic mice were assayed by ELISA. The content of the cytokines per mg brain tissue was calculated for statistical analysis. One way ANOVA with LSD post-hoc test was used for statistical analysis (*n* = 6). **E.** The serum GDF11 level of APPswe/PSENldE9 transgenic mice with SCs treatments was assayed by ELISA. One way ANOVA with LSD post-hoc test was used for statistical analysis (*n* = 6). **F.** The GDF11 expression of spleen isolated from old APPswe/PSENldE9 transgenic mice (14–15 month-old) and young mice (young, 6–8 week-old) mice was compared. Spleen homogenates were used for assaying the GDF11 expression in spleen by ELISA (Student's *t*-test for statistical analysis). Data are presented as mean ± SEM, *n* = 5, *p < 0.05.

Research has shown that immune response and cytokine expression in the brain contribute to or prevent AD progression [29–31]. Splenocyte administration may affect brain function and AD pathology through the regulation of cerebral cytokine expression. Therefore, we used ELISA to measure the levels of IFN-γ, IL-2, IL-4, IL-10 in the brains of aged APPswe/PSENldE9 mice after splenocyte administration (Figure 6A–6D). Neither administration of old or young splenocytes affected cytokine levels in the brains of APPswe/PSENldE9 mice.

To explore how the splenocyte treatments exerted their effects in AD, we assayed GDF11 expression in mice serum with ELISA. We found that ySCs treatment significantly increased systemic GDF11 levels compared with control (One-Way ANOVA with LSD post-hoc test, *F*_(2, 13)_ = 2.812, *p* = 0.034; Figure 6E). However, there was no difference in GDF11 expression in the spleens of young WT mice and old APPswe/PSENldE9 mice (14–15 month-old) (Student's *t*-test, *t* = 1.462, df = 8, *p* = 0.445; Figure 6F). The source of the increased systemic GDF11 levels in old AD mice with ySCs treatment is yet to be determined.

## DISCUSSION

In this study, we transplanted young WT mouse-derived splenocytes (ySCs) into old APPswe/PSENldE9 transgenic mice to ameliorate AD pathology. Our results showed that ySCs not only reduced cerebral Aβ burden and decreased neuroinflammation, but also contributed to the improved spatial learning and memory of APPswe/PSENldE9 mice, whereas oSCs (splenocytes derived from old transgenic mice) increased cerebral Aβ burden, boosted neuroinflammation and worsened cognitive deficits. Our studies showed that ySCs increased systemic GDF11, which was demonstrated to contribute to neurogenesis and cognitive improvement [25]. At the same time, an increased frequency of Treg cells in oSCs was also found, which implies involvement in the increased deficits in spatial learning and memory seen in mice with oSCs treatment.

Our study showed that ySCs treatment decreased cerebral amyloid plaques. This demonstrates that young splenocytes enhance Aβ clearance in the brain of old AD mice. However, transplanting oSCs increased cerebral Aβ burden, indicating that splenocyte senescence impacts Aβ pathology in AD progression [32]. This result shows that immunosenescence is not only limited to the effect on Aβ clearance, but is also involved in Aβ pathology. Therefore, reversing immunosenescence is a potential target for preventing Aβ pathology [33].

As our previous study showed, CD68 positive microglia were able to phagocytose Aβ more effectively in old APPswe/PSENldE9 mice [26]. In this study, all the treatments with splenocytes did not significantly increase the Iba-1 positive microglia in the brains of old AD mice. It was the treatment with oSCs, but not ySCs, that enhanced cerebral CD68 expression. Increased CD68 positive microglia could not increase Aβ clearance, but instead accelerated the deposit of Aβ. Moreover, excessive glia response as a proinflammation factor in the brains of AD mice accelerated AD pathogenesis [34]. The ySCs treatment decreased astrocytic response, contributing to the prevention of AD pathogenesis and the improvement of cognitive function [3]. Our data implies that higher astrocytic response and increased CD68 positive microglia in mice treated with oSCs results in an increased inflammatory response in the brain and thus exacerbates Aβ burden and decreases cognitive function. These findings are consistent with previous reports that immunosenescence results in over-inflammation and restricts therapeutic benefit for AD mice [3, 12, 15, 35].

Morris Water Maze tests indicate that ySCs are more beneficial for the improvement of cognitive function in AD mice. This was also supported by other studies [24, 25, 36–38]. Although ySCs treatment could not significantly improve spatial learning and memory compared with PBS control, there was an obvious decrease in cognitive impairments in the old AD mice compared with the oSCs treatment. This corresponds to the results that ySCs treatment did not reduce amyloid burden in the hippocampus, an area responsible for spatial learning and memory.

Moreover, we found oSCs treatments increased deficits in spatial learning and memory, which supports the idea that immunosenescence can accelerate pathological process and cognitive dysfunction [39, 40]. This also suggests that aged splenocytes are associated with cognitive impairment in old AD mice [37, 39, 40], which may counteract the benefits of ySCs treatment for decreasing cognitive impairment. To test this, we identified that ySCs treatment could neither completely replace the senescent immune system, nor significantly prevent cognitive decline of old AD mice, although it was able to significantly reduce cerebral Aβ burden and decrease neuroinflammation. This also suggests that a complete reversal of immunosenescence should be more effective in the prevention of AD progression and cognitive decline.

It has been reported that the improved cognitive function of AD mice was in line with the regulation of synaptic plasticity, increased neurogenesis and the density of synapse [7, 36], However, in this study, we did not find any difference in synapse morphology or levels in the brains of AD mice in all treatment groups.

Regulatory T cells (Tregs) play important roles in both the immune system and the nervous system. Some studies reported Treg cells as a neuroprotective immuno-modulator in AD [38, 41, 42]. However, our study suggests that old AD mice derived Tregs might have had a detrimental effect on AD pathology and cognitive function in AD mice. We did not detect any difference in the percentage of CD3^+^ T cells and CD4^+^ T cells in SCs between young WT mice and old AD mice. Of note, we found the proportion of Treg cells in splenocytes was increased in old AD mice. We considered the increase in Treg cells to be associated with immunosenescence, which is consistent with a recent report [43]. Moreover, the increase in Tregs could explain why AD mice with oSCs treatment had increased neuroinflammation. This also coincides with other findings that Tregs of oSCs cells were dysfunctional, or that the pro- inflammation cells were the major cells in oSCs [44].

Both splenocyte treatments altered microglia and astrocytic response in brains of APPswe/PSENldE9 mice, however the cerebral levels of IFN-γ, IL-2, IL-4, IL-10 did not correlate with microglia and astrocytic response These cytokines were considered to be the key cytokines affecting microglia and astrocytic response in brain [30, 45], but, our results suggest that these cytokines are not closely associated with changes of microglia and astrocytes in brains of APPswe/PSENldE9 mice after splenocyte treatment.

It was recently demonstrated that systemic GDF11 reversed senescence and improved cerebral vasculature, enhancing neurogenesis in old mice [25, 46]. We proposed that young splenocytes may ameliorate neurodegeneration via anti-aging factors. In order to explore this, we analyzed the expression of GDF11 both in serum and spleen of AD mice. We found that ySCs treatments increased GDF11 levels in AD mice blood. This implied that the elevated systemic GDF11 level in old AD mice might contribute to the ameliorative effect on cognitive decline. However, the source of the increased systemic GDF11 needs to be explored. Possibly, some of the transplanted ySC cells enhanced GDF11 expression in old AD mice, or perhaps some of the transplanted ySCs residing in old AD mice directly secreted the factor and elevated the systemic GDF11 levels.

Our study shows that ySCs administration is beneficial for APPswe/PSENldE9 transgenic mice, whereas oSCs administration increased cerebral Aβ burden, boosted neuroinflammation and increased cognitive deficits. This demonstrates that oSCs administration is detrimental for AD mice. Moreover, oSCs also had an increase in Tregs and were unable to elevate the systemic GDF11 levels compared with ySCs. Both Alzheimer's disease and aging can influence the functions of splenocytes, and thus result in the detrimental effects of oSCs [39, 43, 47–49]. However, at present we cannot determine which factors result in the detrimental effects, further studies are needed.

In summary, the findings in this study imply that rejuvenating the immune system by inhibiting immunosenescence represents a promising therapeutic strategy for AD. The related immunological mechanism requires detailed exploration. Hopefully, this will encourage others to further investigate this new immunological treatment for AD and other age-related diseases.

## MATERIALS AND METHODS

### Animals

APPswe/PSENldE9 transgenic mice expressing a mutant human presenilin 1 carrying the exon-9-deleted variants (PSENldE9) and a chimeric mouse/human amyloid precursor protein (APPSwe, Swedish mutations K595M/N596L) [50] were contributed by the Neurobiology and Genetics Laboratory of the Rockefeller University and reproduced in the animal lab of Fudan University. All experiment procedures and animal care were in compliance with the regulations of Fudan University for animal experimentation and the NIH guidelines for the Care and Use of Laboratory Animals.

### Splenocytes collection

Splenocytes were isolated from dissected spleen of old APPswe/PSENldE9 transgenic mice (14–15 months) or young non-transgenic mice (6–8 week-old APPswe/PSENldE9 transgenic littermates). Isolated spleens were crushed in sterilized phosphate buffered saline (0.01 M PBS) and through 100-μm nylon cell strainer (BD Falcon, USA) to obtain single cell suspension. Cells were collected and washed with PBS for following administration and assay.

### Flow cytometry

Single-cell suspensions of splenocytes were prepared for flow cytometry. Anti-CD16/CD32 (eBioscience, USA) was used to block Fc receptors. T cells were stained with PE anti-mouse CD3 IgG (BioLegend, USA) and FITC anti-mouse CD4 IgG (eBioscience, USA). Treg were stained with Mouse Regulatory T Cell Staining Kit (eBioscience, USA) as following. Cells were incubated with FITC anti-mouse CD4 IgG and PE anti-mouse CD 25 IgG for 30 min at 4°C. For staining the intracellular Foxp3, cells were treated with Fixation/Permeabilization solution for 2 h at 4°C, stained with PE-Cy5 anti-mouse/rat Foxp3 antibody. After washing, cells were analyzed using Flow Cytometer (Beckman Coulter, USA).

### Splenocytes administration

Sterilized single-cell suspension of splenocytes (5 × 10^7^/ml splenocytes in 0.01M PBS) was used for the administration. Male APPswe/PSENldE9 transgenic mice (14–15 months) received intraperitoneal injections of 200 μl splenocytes derived from young WT mice (ySCs group, *n* = 6), or splenocytes derived from old APPswe/PSENldE9 transgenic mice (oSCs group, *n* = 6), or 0.01 M PBS (Control group, *n* = 6). Every mouse received 3 times injections on day 1, day 14, and day 45 (shown by Figure 1A). Splenocytes were prepared just before every injection. One week after the third injection, Morris water maze was performed, then Open field and Step down two weeks later.

### Behavior assays

#### Morris water maze (MWM)

MWM was performed in a circular pool of 150 cm diameter with a 40 cm high wall in a quiet room at 22 ± 1°C as previous described [51]. The pool filled with 20 cm deep opaque water was divided into four equal quadrants according to a MWM system (ANY-maze Version 4.84, Stoelting Co.). All mice were trained to find a 10- cm-diameter escape platform submerged 1 cm under water for 6 days, 4 training trail per day. In the training trials, when mice found and climbed up the platform in 60 seconds and remained for 15 seconds, the escape latency was recorded. The escape latency in 6 days was analyzed to evaluate spatial learning and memory ability. After 6 days training trails, the spatial probe test was performed. The escape platform was removed, and each mouse was individually allowed to swim freely for 60 seconds. The swim path of every mouse was recorded by a video camera connected to a personal computer, suspended above the pool.

#### Open field

Open field test was performed in a chamber of 50 cm × 50 cm × 50 cm. Each mouse was placed in the open field, and the vertical and horizontal movement in 20 minutes was recorded for analysis.

#### Step-down

Step-down test was performed in a chamber of size 30 cm × 30 cm × 30 cm, the floor was laid with electrical circuit connected to a 50V source according to previous description [26]. A platform of 10 cm × 10 cm was located at a corner on the floor. On 1^st^ day, each mouse was placed on the platform. The latency period of stepping down the platform was recorded as the baseline time. Switching the current on, mice were placed on the platform. Upon stepping on the floor, mice will get a foot shock. The latency period of stepping down platform after the foot shocking was recorded. 24 hours and 48 hours after foot shocking, mice were placed on the platform, and the latency period of stepping down was record.

### Tissue samples and blood collection

Tissue samples and blood were collected after behavioral test. After euthanizing, blood was collected respectively by right ventricular puncture from every mouse. Each mouse was perfused transcardially with 0.9% NaCl immediately after bleeding. Brain was removed and divided into two hemispheres by cerebral longitudinal fissure. One hemisphere was fixed for 12 h in 4% formaldehyde solution, then dehydrated by sucrose solution, embedded in O.C.T. Compound (SAKURA, USA) and frozen at −80°C.

### Tissue lysis and western blot

The frozen hemisphere tissues (cortex and hippocampus) were homogenized and lysed in cold RIPA buffer (50 mM Tris (pH 7.4), 150 mM NaCl, 1% Triton X-100, 1% sodium deoxycholate, 0.1% SDS, sodium fluoride, EDTA, leupeptin, 1 mM sodium orthovanadate and 1 mM Phenylmethanesulfonyl fluoride). Supernates were collected after centrifugation at 14,000g for 30 min. Equal amounts of total protein homogenization were separated on SDS-PAGE gels and transferred onto polyvinylidene difluoride (PVDF) membranes. The membranes were blocked for 2 hour with 5% BSA and incubated with the specific antibodies at 4°C overnight: Rabbit Anti-PSD-95 IgG and Rabbit Anti-Synaptophysin IgG (Cell Signaling Technology, Danvers, MA, USA) were used for synapse detection, Mouse Anti-GAPDH IgG (Kang Chen Biotech Inc., China) was used as a loading control. The appropriate horseradish peroxidase-conjugated (HRP)-conjugated antibodies were incubated for 2 hours at room temperature to detect the specific antibodies. The chemiluminescent signals for proteins were scanned by ChemiDoc™ XRS+ System with Image Lab™ Software (Bio-Rad, Hercules, CA, USA) and analyzed using a Gel-Pro analyzer (Media Cy-bernetics Inc., Silver Spring, MD, USA).

### Cerebral cytokine assay by ELISA

Brain homogenates were used for cerebral cytokines assay. The levels of IFN-γ, IL-2, IL-4, IL-10 in brain were detected with a Th1/Th2 ELISA kit (eBioscience, USA) according to the Corp's manual.

### Immunohistochemistry for brain sections

Series coronal brain sections were cut at 20 μm thick, with a freezing microtome. Sections for DAB (3, 3′-diaminobenzidine) staining were quenched by 3% H_2_O_2_, blocked by 5% serum containing 0.1% Triton X-100, and incubated with primary antibody overnight at 4°C. Aβ was detected by Mouse Anti- Aβ IgG (Sigma-Aldrich); microglia was stained with Rabbit Anti- Iba1 IgG (Wako Pure Chemical Industries) and Rat Anti- CD68 IgG (BioLegend); astrocyte was stained with Rabbit Anti- Glial Fibrillary Acidic Protein (GFAP) IgG (Dako A/S, Denmark). The appropriate HRP-conjugated antibodies were incubated for 2 hours at room temperature, and stained with DAB substrate (Sigma-Aldrich). Synapse was detected by Rabbit Anti- PSD-95 IgG (Cell Signaling Technology, Danvers, MA, USA) and Goat Anti- MAP2 IgG (Santa Cruz, California, USA). Images were acquired using a CCD camera on an Olympus BX70 microscope connected to a computer. For analysis, Images of 9 sections per mouse were captured with 10× and 20× objectives and assessed by NIH image J. The whole region of cortex and hippocampus in every brain sections was selected for images analysis. Amyloid plaques and CD68 positive granules were defined by threshold setting with NIH image J system. The percentage of amyloid covered areas on the cortex or hippocampus region was used for statistical analysis. Iba1 and GFAP positive cells were also counted with NIH image J system. The density of the cells (cells per mm^2^) in the region of cortex and hippocampus was calculated.

### GDF11 assay by ELISA

Blood was allowed to clot for 2 hours at temperature, and centrifuged for 20 minutes at the speed of 1000 × g. Serum was collected for GDF11 assay. Spleen was isolated from mice and homogenized with ice-cold PBS (50 μl 0.01 M PBS per 10 mg of spleen), then centrifuged for 5 minutes at the speed of 5000 × g. The supernatant was removed to assay the expression of GDF11 in spleen. The GDF11 levels of serums and spleen homogenates were assayed by ELISA with a GDF11 ELISA kit (Cloud–Clone Corp., USA) according to the Corp's manual.

### Statistical analysis

Date is expressed by mean with standard error (SEM). Statistical differences were examined using Student's *t*-test, one way analysis of variance (ANOVA), repeated-measures ANOVA, or Kruskal-Wallis non-parametric ANOVA by SPSS19 software. *p* < 0.05 was considered as significantly different.
